# The evaluation of individuals under the age of 18 working at Şahinbey automotive industrial site in Gaziantep province

**DOI:** 10.3389/fpubh.2024.1484785

**Published:** 2024-11-22

**Authors:** Coşkun Cüce, Neriman Aydın

**Affiliations:** Faculty of Medicine, University of Gaziantep, Gaziantep, Türkiye

**Keywords:** child labor, Gaziantep, automobile industry, occupational health, safety

## Abstract

**Introduction:**

Child labor is an important issue of public health in both developed and developing countries. This is a cross-sectional-descriptive study.

**Methods:**

418 individuals under the age of 18 were enrolled in the study. Data were collected by using a questionnaire. The data were analyzed using SPSS 22 software package. Significance was *p* < 0.05.

**Results:**

All the participants were male with a mean age of 14.90 ± 1.94 years. 34.9% did not continue education. Among them, prevalence of underweight, shortness, overweight and obesity were 3.3, 9.9, 17.7 and 6.2%, respectively. 16.5% of the families had immigrated. The mean number of family members was 5.87 ± 1.62. Out of the participants, 29.4% were smokers, 8.4% had alcohol abuse and 3.8% had substance abuse. 55.0% of the children stated to have started working for acquiring a profession, whereas 35.4% were for financial reasons. The children were working mostly at automobile repair shops (38.8%). The average working time was 11.46 ± 0.83 h per day. 5.7% of the children were attending apprenticeship school. 52.6% said they had at least one occupational injury event.

**Discussion:**

It was found that age, reason for working, the degree of proximity to the employer, and having a master trainer’s certificate of the employer affected the children’s attendance at Apprenticeship Training Center. Occurrence of having occupational injury events was found to be associated with sleep duration, experiencing abuse, smoking and alcohol addiction. Child labor still remains unsolved despite the measures taken and the long struggle. It will be useful to support them in healthy life behaviors such as healthy sleep, healthy nutrition, no smoking and alcohol habits. More radical solutions should be sought to eliminate child labor.

## Introduction

According to Article 1 of the United Nations (UN) Convention on the Rights of the Child, every human being up to the age of eighteen is considered a child, except in the case of an earlier age of majority (1). The International Labor Organization (ILO) defines child labor as any kind of work that hampers the child’s health, education, physical and mental development, as well as depriving them of their potential and dignity. The Report on Child Labor published in 2013 specifies a decrease in the number of child laborers across the world between 2000 and 2012. However, it has stated that currently 168 million children continue to work, and that 85 million are still working in dangerous conditions, corresponding to more than half of them (2). The studies suggest that social, economic and political factors play an important role in the employment of children, with family poverty being the main determinant factor (3–5). Child labor has serious negative effects. It is known that engaging in economic activities at an early age has destructive effects on physical and mental development. The children who have to work by leaving their childhood aside as well as giving up their education are faced with many additional problems such as poor working conditions, inadequate wages, high-responsibility tasks, occupational injury events, stress, exposure to physical, emotional or sexual abuse (6). Protecting the child laborers against any kind of negligence and abuse, and ensuring them legal and social protection as well as preparing them for the future are all important aspects in terms of both community development and human rights (7). Therefore, the most important components of such interventions should comprise increased support to both education and families (8).

The aim of the study is to determine the socio-demographic characteristics, working conditions, general health status, habits and substance abuse status, the current issues experienced by child laborers in the auto industrial industry to provide information that can be used in policies and interventions.

## Subjects and methods

This study is a descriptive cross-sectional study. All individuals under the age of 18 who have worked at Şahinbey Automotive Industry Site in Gaziantep province, and accepted to participate were included in the study (*n* = 418). Verbal consent was obtained from the children and one of their parents for the study. The questionnaire prepared with the literature review was applied to the children by the researchers through face-to-face interviewing. The questionnaire included questions about the socio-demographic characteristics of the children, information about their working conditions, and the occupational injury events. After the questionnaire was prepared, a pilot study was conducted with 15 children and the questionnaire was finalized. In addition, the height and weight of individuals under the age of 18 were measured during the study by the researchers. Weight measurement was performed on a flat ground using a digital weighing instrument having a precision of 0.1 kg, with children’s shoes being taken off. The height measurement was performed barefoot between the sole and the crown with a precision of 1 cm in upright position, using a thin bar parallel to the floor in contact with the head. Body Mass Index (BMI) (kg/m2) was calculated for each child. The WHO 2006 Child Growth Standards were employed in the comparisons (9). Accordingly, BMI for age < −2 standard deviations was considered underweight and > +2 standard deviations was considered obese. Height-for-age < −2 was considered stunted.

Prior to the study, approval was obtained from Gaziantep University Clinical Research Ethics Committee. The obtained data were analyzed by SPSS (Statistical Package for the Social Sciences) version 22 statistical package program. Chi-square analysis was used along with descriptive statistics. The level of significance was accepted as 0.05.

## Results

All of the child laborers enrolled in the study were male, with a mean age of 14.9 ± 1.9 (ranging from 8 to 17 years), and 37.3% were under the age of 15. While 91.6% of the participants were Turkish citizen, 8.4% were citizens of Syria. 22.5% of the children had no health insurance. 34.9% of them were found to have given up their education. As the underlying reasons for non-attendance at school, 58.9% of them were not successful to pass their courses, while 25.3% were deprived of financial support. It was shown that the children living with 1 to 5 family members had a significantly higher level of commitment to education as compared to those living with 6 or more family members, which is also true for the comparison of those with at least a medium level of wealth versus those with poverty (*p* < 0.05). It was determined that 28.5% of participants were living in slums, and 84.4% were sharing their home with 5 or more family members.

For the child laborers, 72.7% of mothers and 59.6% of fathers had primary education at the most. 10.0% of fathers and 97.4% of mothers were unemployed. Out of working fathers, 48.8% were laborers, 22.7% were automotive industry workers, 22.2% were shopkeepers, and 6.2% were either farmers, civil servant or retired. 16.5% of the children’s families had migrated to Gaziantep from another settlement. It was found that 50.8% of these families had migrated to escape from war, and 37.7% for financial reasons.

85.9% of the child workers stated that they had breakfast every day, and 5.3% did not have any breakfast. For the lunchtime, 92.4% of the children stated that they were eating fast foods, while 6.2 and 1.4% were eating daily food menu prepared at workplace and foods either from restaurants or their homes, respectively. Among the participants, it was found that 9.9% were stunted, 65.1% had normal height, 3.1% were thin, 72.8% had normal weight, and 6.2% were obese.

According to the statements of child workers, 28.0% were smoking, 8.4% had alcohol use, and 3.8% were addicted to substance (Table 1). For those who had substance abuse, 50.0% were addicted to marijuana, 37.5% were addicted to crystal meth, 18.8% had glue sniffing and paint thinner addiction, and 18.8% had drug addiction.

**Table 1:** Distribution of child laborers by smoking, alcohol and substance abuse.

Variables		Number (n)	Percent (%)
Smoking	Yes	117	28.0
	No	301	72.0
Alcohol use	Yes	35	8.4
	No	383	91.6
Substance abuse	Yes	16	3.8
	No	402	96.2
	Total	418	100.0

Table 2 shows the lines of work that participants were engaged. While 85.4% of the children started working below the age of 15, 14.6% went out to work at or over 15 years old. The average age of onset for working was 11.8 ± 2.5 years (ranging from 5 to 17). 31.5% of the children stated to have worked unwillingly. It was established that 55.0% were working for gaining an occupation, 35.4% for financial reasons, and 28.7% for not being unemployed. The average working hour of the child laborers excluding the breaks was found to be 11.5 ± 0.8 per day. The number of days child laborers worked in a week was different. It was stated that 99.8% of the child laborers worked at least 8 h per day and 43.3% were run errands (Table 3). The average weekly incomes were found to be 98.9 ± 57.8 Turkish Liras (TL) (26.1 American Dollars)., and 1.0% worked without any incomes. The rate of main tasks assigned to those aged 15 or above and their weekly incomes were found to be significantly higher (*p*-value <0.05).

**Table 2:** Distribution of child laborers by lines of work.

Line of work	Number (n)	Percent (%)
Car repair	159	38.0
Auto electrics and electronics	56	13.4
Auto bodywork	56	13.4
Auto painting	25	6.0
Auto lathe	25	6.0
Auto spare parts	19	4.5
Auto tires	12	2.9
Other*	66	15,8
Total	418	100.0

*Comprising child laborers working in auto upholstery, engine cover repair, auto accessory, LPG installation and repair, exhaust line, coil winding, restaurants, radiator repair, car wash, auto scrappage, auto appraisal, wheel balancing, auto oiling, car glass, bumper repair, confectioner and bagel selling, barber shop, and grocery.

**Table 3:** Daily working hours of the child laborers excluding the breaks, types of work, and distribution of their weekly incomes.

Variable		Number (n)	Percent (%)
Daily working hours excluding the breaks	Under 8 h	1	0.2
	8 h or longer	417	99.8
Types of work that are mostly involved	Running errands	181	43.3
	Assisting	172	41.2
	Cleaning	41	9.8
	Main tasks	24	5.7
Total incomes corresponding to their weekly workload	0–50 TL (0–13.2 American Dollars)	104	24.9
	51–100 TL (13,3–26,4 American Dollars)	174	41.6
	101 TL and more (26,5 American Dollars and more)	140	33.5
	Total	418	100.0

27.5% of the child laborers arrived to workplace through their own means, while 72.5% used transportation facility provided by employers. 95.7% of them declared that they were satisfied with their work. Of those unsatisfied with their work, 77.8% attributed their dissatisfaction to lower wages, 16.7% to heavy working conditions, and 5.6% to unfavorable working hours.

The statements of child laborers have shown that none of them had a periodical health examination nor have been vaccinated against tetanus prior to start date of employment. It was also found that all the children were made to work on some national holidays.

It was reported that 52.6% of the children were exposed to occupational injury events, 21.4% of which led them give up working for a while, and 0.5% of which caused them to leave or change their jobs. The most common types of occupational injury eventss included cuts and punctures (57.1%), burns (16.9%), crushing and bruises (8.3%), and fracture and dislocation (7.1%) (Table 4).

**Table 4:** Distributions for occupational injury events of child laborers and the impacts of occupational injury events on working by the types of injury.

Variable		Number (n)	Percent (%)
Occurrence of having occupational injury events	Having any occupational injury event	220	52.6
	Having no occupational injury event	198	47.4
	Total	418	100.0
occupational injury event on working	Not affected	172	78.2
	Those could not resume work for a while	47	21.4
	Those who had to leave or change their work	1	0.4
	Total	220	100.0
injury event	Cuts and Punctures	145	57.1
	Burns	43	16.9
	Crushing and Bruises	21	8.3
	Fractures and Dislocations	18	7.1
	Other	27	10.6
	Total*	254	100.0

*A number of child laborers declared to have been exposed to more than one occupational injury event.

5.7% of the child laborers reported that they were attending the Apprenticeship Training Center (ATC). Regarding to the statements of apprentices on explaining the reason for attending the Apprenticeship Training Center, 91.7% referred to obtaining journeyman’s or mastership certificate, and 8.3% for improving themselves in their occupation. A significantly higher commitment to the ATC was observed among those who were at or above the age of 15, who were not attending at school, who worked primarily for gaining an occupation, who had a foreign employer and whose employer had qualified instructor certificate (*p*-value <0.05) (Table 5).

**Table 5:** Factors affecting studying at the apprenticeship training center.

Factors affecting attendance at the apprenticeship training center		Studying at the apprenticeship training center					
		Continued		Non-continued		Total	*χ*^2^ *p*-value
		Number (*n*)	Percent (%)	Number (*n*)	Percent (%)	Number (*n*)	
Age group	<15	0	0	156	100.0	156	15.161 0.000
	≥15	24	9.2	238	90.8	262	
Continued education status	Continued	8	2.9	264	97.1	272	11.285 0.001
	Non-continued	16	11.0	130	89.0	146	
Main reason for work	Learning a profession	19	8.3	211	91.7	230	5.997 0.014
	Other	5	2.7	183	97.3	188	
Degree of proximity to the owner	Acquaintance/relative	10	3.6	268	96.4	278	7.053 0.008
	Foreign	14	10.0	126	90.0	140	
Master trainer certificate at the employer	Yes	17	9.2	168	90.8	185	7.289 0.007
	No	7	3.0	226	97.0	233	

Those sleeping less than 7 h a day, smokers, alcohol users and those abused at work were significantly more likely to have occupational injury events (*p*-value <0.05). No significant effect of age group, attendance at school, BMI by age, substance abuse, daily working hours, starting and leaving hours for work were found on occupational injury events for the children (*p*-value >0.05) (Table 6).

**Table 6:** Some of the factors affecting occupational injury events.

Factors affecting occupational injury events		Occupational injury events					
		Yes		No		Total	*χ*^2^ *p*-value
		Number (n)	Percent (%)	Number (n)	Percent (%)	Number (n)	
Age group	<15	79	50.6	77	49.4	156	0.396 0.529
	≥15	141	53.8	121	46.2	262	
Continued Education Status	Continued	140	51.5	132	48.5	272	0.421 0.516
	Non-continued	80	54.8	66	45.2	146	
BMI by age	Underweight	8	57.1	6	42.9	14	3.248 0.197
	Normal	194	51.3	184	48.7	378	
	Obese	18	69.2	8	30.8	26	
Smoking	Yes	77	65.8	40	34.2	117	11.232 0.001
	No	143	47.5	158	52.5	301	
Alcohol use	Yes	31	88.6	4	11.4	35	19.791 0.000
	No	189	49.3	194	50.7	383	
Addictive Substance Use	Yes	10	62.5	6	37.5	16	0.650 0.420
	No	210	52.2	192	47.8	402	
Sleep Duration	<7 h	90	62.5	54	37.5	144	8.581 0.003
	≥7 h	130	47.4	144	52.6	274	
Daily working hours	<8 h	17	54.8	14	45.2	31	0.065 0.798
	≥8 h	203	52.5	184	47.5	387	
Starting time of work	08.00 a.m. and earlier	171	52.0	158	48.0	329	0.267 0.606
	After 08.00 a.m.	49	55.1	40	44.9	89	
Time of leaving work	08.30 p.m. and earlier	200	52.1	184	47.9	384	0.569 0.451
	After 08.30 p.m.	20	58.8	14	41.2	34	
Experiencing abuse	Yes	205	55.3	166	44.7	371	8.887 0.003
	No	15	31.9	32	68.1	47	

## Discussion

Child labor is an unacceptable issue under any circumstances. The employment of children under harsh conditions without social care, not only leads to physical, psychological, social and financial damages, but also harms their future.

37.3% of the children are employed under the age of 15 years, which is illegal in accordance with the Labor Code (10). Furthermore, it was found that such children had no insurance by their employers. Therefore, employers are able to fire them arbitrarily, and they have no employment security. This is one of the reasons that employers prefer child laborers. In addition, it is not possible to insure legally for those under the age of 15 years.

As in our study, similar studies on child labor conducted in our country have shown that a wide range of the children from 32.9 to 78.7% continue their education (11, 12). It has been found that child workers were deprived of the right for education and training at varying rates. If child laborers are not enabled to continue education, it will be inevitable to raise an unskilled or low-skilled generation in the future. Consistent with our study results, it has been reported that 40 to 50% of the children left their school prematurely due to lack of interest and failure, whereas 20 to 37.5% for financial reasons (11, 13, 14). If the problems of poverty and social inequality are solved, most of the children are believed to be willing for resuming their education.

Consistent with our findings, other similar studies have shown that the families of child laborers were crowded with more than one child (15–17). According to the 2013 Turkish Demographic and Health Survey (TNSA) data, the average number of persons per household was reported to be 3.6 in Turkey (18). However, it was found to be 5.8 in our study. Since the limited income is shared by many individuals as a consequence of the nature of a crowded family, more individuals have to contribute to family income, which is thought to push children to work.

According to the data from Turkish Statistical Institute (TURKSTAT), the proportion of those graduated from at least high school or their equivalents in 2013 to the total population are 24.3% for males and 17.7% for women in Gaziantep; however, our study has shown that only 5.2% of mothers and 10.5% of fathers have graduation from these schools (19). It was found that the education level of the parents were quite lower, the majority of whom had primary education level at most. This is considered one of the reasons pushing the children to work. According to the data from TURKSTAT, the rate of education level at or above high school in working parents is well below the average of Turkey. Our study has demonstrated that, 10.0% of the fathers and 97.4% of the mothers did not work. Similar to other studies, the fathers were found to be rather working as laborers, shopkeepers or farmers (12, 20). It is known that parental unemployment and working in the marginal sectors are important reasons for children to work.

Consistent with our study, similar other studies have also shown that 7.5 to 35.9% of the families of child laborers had migrated (15, 20). In a similar study, work-related migration was found to be on the first rank (13). Our study has shown the first reason for migration as escaping the war. In the studies, it was determined that the causes of migration were mainly due to work, but different reasons were come into prominence in different geographical areas. The war in Syria, a neighboring country of Turkey, ranks the first for reasons of migration.

Our study has demonstrated that 9.9% of child laborers were stunted, 3.3% were thin, 17.7% were overweight, and 6.2% were obese. Researches on child laborers has revealed that 14.4 to 23.3% were underweight, 3.9 to 15.6% were overweight, and 1.6 to 3.8% were obese (12, 20). According to the results of the 2010 Turkey Nutrition and Health Survey (TNHS), in those aged between 6 and 18 years, the proportions for shortness, underweight, overweight and obesity were 6.8, 3.8, 14.2 and 7.3%, respectively (21). We have found a lower frequency of underweight and a higher frequency of obesity in our study, as compared to the data from other studies on child laborers. It is thought that the reason would be the fact that 92.4% of the children consume unhealthy and high-calorie fast-foods every day. The higher consumption rate of fast-food may result from the fact that the children can consume this type of food at a fast pace due to lack of enough time, they think that fast-foods are more delicious, or the lack of restaurants serving stew meals at the time of data collection. In our study, the frequency of overweight and obese individuals was similar to those of TBSA results; however, it was higher regarding the frequency of shortness, which was thought to have association with heavy working conditions. In addition, the rates for smoking, alcohol and substance abuse were higher in child laborers as compared to other studies conducted on students of the same age (22–24). This may be due to their acceptance of the employees in the workplace as role models, or they may related to negative peer influence in the workplace.

In a number of studies conducted on automotive industrial sites either in Turkey or other countries, it has been shown that child laborers primarily work in auto repair shops, followed by mostly in bodyworks, auto electrics, auto painting and lathe workshops, which are consistent with our results as well (11, 12, 16, 25). The reason why child laborers mostly work in auto repair shops may be that more repair kits used there and children are used to carry this materials. While similar studies in Turkey have also shown that the primary reason for children to work is to gain an occupation, studies from other countries have shown financial reasons to be more prominent (11, 16, 26). Our study has found that the majority of child laborers are employed in subtasks rather than main tasks, the proportion of those assigned to main tasks increases as they get older, and those assigned to main tasks have higher weekly income. It was also assumed that incomes could be lower for subtasks, thus leading to a lower level of occupational satisfaction. A higher rate of those engaged in main tasks with increased age was associated with the current state of their knowledge as well as establishing trust between master and apprentice. In our study, the lower rate of those engaged in main tasks was related to the fact that a substantial part of children were casual laborer.

Our study has shown that the average working hour of child laborers was 11.5 ± 0.8 per day excluding the breaks, and those working longer than 8 h per day were 99.8%. In studies conducted on child workers, it has been observed that the average working hour ranged between 10.6 and 11.3 per day, with the rate for working longer than 8 h ranging from 47 to 95.1% (11, 13, 14, 27–34). All the children in our study were found to exceed the legal limits according to the Labor Code and to work longer as compared to the results of other studies. It can be seen that the weekly incomes of child laborers were lower, which is thought to be due to the fact that the majority of them did not sign an apprenticeship contract, they had no knowledge about their rights, and that the audits were not adequate. It was also established in our study that all of the child laborers worked during some public holidays. The Regulation on the Principles and Procedures for the Employment of Children and Young Persons sets forth that child and young workers should not be employed on national festivals and public holidays, and their corresponding incomes for these days should be paid to them (35). Such an exceeding of legal working hours and lower wages clearly reveal labor exploitation. It is thought that these severe working conditions may lead to physical and mental disorders as well as excessive fatigue in children. It should be noted that the lack of pre-employment and periodic health examinations may cause the children to work in jobs not suitable for their health, body size or skills, and their current health problems to deteriorate that could otherwise be prevented if diagnosed earlier.

While several studies has found that 21.1 to 87.6% of the child laborers were attending at the apprenticeship school, this proportion was only 5.7% in our study (11, 13, 31, 36). These higher rates of attending to the ATC as compared to our study was thought to result from the fact that most of data were collected in summer season, and that as many as 60.9% of those not attending to the ATC were casual laborers. It is anticipated in our study that the children at or above the age of 15, those not attending at school, and those with their employers having qualified instructor certificate should be enrolled in the ATC, and those who have already enrolled should display much commitment to the ATC because of benefiting from health insurance. Getting into working life at their own request and aiming primarily to gain an occupation suggest that the children have future career plans. The fact that the documents required to start up a business can be obtained from the ATC is thought to be the reason for the children in their attendance to the ATC.

Studies has shown that a wide range of the child laborers between 34.0 and 71.4% had occupational injury events, the range of which remained the same in our study as well (11, 16, 37, 38). Likewise, it also has been shown in our study that the most common occupational injury events for the children included cuttings, punctures, burns and fallings (16). Unlike our study, İşeri et al. have found that those getting into work life under the age of 15 had more occupational injury events (39). In line with our study, Akyan and Atak have shown that age groups, presence of social security, type of work and daily working hours have no impact on having an occupational injury event (11). It was found in our study that those sleeping less than 7 h, smoking, taking alcohol and getting punished at workplace had more occupational injury events. It is known that insufficient sleep is likely to cause sleepiness, which is also true for alcohol use leading to fatigue and attention deficit. The children having bad habits and getting punished were considered to be nervous and non-adaptive, which thus may lead to increased rate of injury events. Age and daily working hours were found to have no effect on the rate of occupational injury events. This may have been due to the fact that all children participating in the study were already at an age when they should not work. The fact that there was no statistically significant difference between working hours and occupational injury events may be due to the fact that working hours were similar for all children in the study.

Child labor, despite the struggle and efforts, increasingly continues to be an international problem on the basis of poverty and income inequality. Family poverty is also among the reasons why children work. Children give the income they earn to their families. Solutions for the general economic situation will be effective in reducing child labor. Children who have to work precariously in heavy conditions with lower wages they may face many physical, mental and social problems in the future. The main goal should be to prevent children from working and to ensure that they continue their education. Children who are able to work within legal limits should be supported in healthy life behaviors such as healthy sleep, healthy nutrition and no smoking and alcohol addiction. Therefore, it can be said that a multi-sectoral cooperation is needed in order to prepare a good future for children by putting them away from working and canalizing into school. Studies revealing the current situation may lead the way in this regard.

## Data Availability

The raw data supporting the conclusions of this article will be made available by the authors upon reasonable request.
